# Highly Stretchable All-Rubber-Based Thread-Shaped Wearable Electronics for Human Motion Energy-Harvesting and Self-Powered Biomechanical Tracking

**DOI:** 10.1186/s11671-019-3085-9

**Published:** 2019-07-23

**Authors:** Jie Zhu, Xinghui Wang, Yilan Xing, Jianyi Li

**Affiliations:** School of Computer and Remote Sensing Information Technology, North China Institute of Aerospace Engineering, Langfang, 065000 China

**Keywords:** Triboelectric nanogenerator, High stretchability, All-rubber-based thread, Biomechanical energy harvester, Self-powered sensor

## Abstract

The development of stretchable smart electronics has attracted great attentions due to their potential applications in human motions energy collection systems and self-powered biomechanical tracking technologies. Here, we present a newly stretchable all-rubber-based thread-shaped triboelectric nanogenerator (TENG) composed of the silver-coated glass microspheres/silicone rubber as the stretchable conductive thread (SCT) and the silicone rubber-coated SCT (SSCT) as the other triboelectric thread. The stretchable all-rubber-based thread-shaped TENG (SATT) generates an open-circuit voltage of 3.82 V and short-circuit current of 65.8 nA under the 100% strain and can respond to different finger motion states. Furthermore, the self-powered smart textile (SPST) woven by the SCT and SSCT units has two kinds of working mechanisms about stretch-release and contact-separation modes. The stretching-releasing interaction between knitting units can generate an open-circuit voltage of 8.1 V and short-circuit current of 0.42 μA, and the contacting-separating mode occurs between cotton and two types material outside the SPST producing peak voltage of 150 V and peak current of 2.45 μA. To prove the promising applications, the SPST device is capable to provide electrical energy to commercial electronics and effectively scavenge full-range biomechanical energy from human joint motions. Therefore, this work provides a new approach in the applications of stretchable wearable electronics for power generation and self-powered tracking.

**Electronic supplementary material:**

The online version of this article (10.1186/s11671-019-3085-9) contains supplementary material, which is available to authorized users.

## Introduction

Wearable electronics with comfort, soft, and breathability integrated on textiles or clothes have been widely used in many fields, such as biomedical monitors [1–3], bionic-robots [4–6], human-interactive interfaces [7, 8], military, and consumer electronics [9–11], which is the perfect embodiment of the boom advancement in technology and brings a lot of convenience and advantages to our life. However, for powering these wearable electronics, traditional batteries and supercapacitors are difficult to meet their energy requirements due to the technical bottlenecks of structural rigidity, limited lifetime, extra device weight, and environmental pollution. Consequently, it is an urgent problem to explore a newly sustainable power supply for wearable electronics. For wearable applications, human motion mechanical energy is ubiquitous and relatively stable that is expected to be converted into electricity by the wearable electronics in operation, developing into a sustainable self-powered multi-functional electronic device [12, 13]. Therefore, it is a promising method for using human motion mechanical energy harvesting technology to achieve a self-powered wearable device, which could convert the measured signals into power supply signals.

Among various approaches, the triboelectric nanogenerators (TENGs) [14–17] base on triboelectric electrification and electrostatic induction can efficiently scavenge human motions mechanical energy, which is regarded as a sustainable power or a self-powered sensor due to lightweight, cost-effectiveness, high efficiency, robustness, and wide selection of materials. Recently, developing thread-shaped TENGs acted as self-powered wearable electronics have been demonstrated their merits in monitoring human physiological signals including body motions detecting, skin tactile sensing, pulse frequency testing, etc. Hongzhi Wang has delivered a thread-like sensor with built-in wavy structure design to detect and discriminate the joint movements of human bodies [18]; however, the stretchability of sensor is a critical hurdle in complex limb motions with large strain. Moreover, the smart textile electronics composed of the thread-shaped TENGs have shown their advantages in human motions energy collection systems owing to easily integrated with clothes. Wang and co-workers have sewn the wearable smart textile into a garment to become a power cloth [19] or realized TENG textiles based on well-designed weaving yarns method [20]; however, the stable high-output performance is still a challenging problem for practical applications. Besides, most stretchable electrodes in previous wearable electronics are achieved by serpentine metal foils [21, 22], deposition on pre-strained soft substrate [23, 24], and metal nanowires [25], obstructing the smart textile electronics to tolerate wearing use and large-scale fabrication.

Here, in order to address the above issues, we present a new type of SATT with double-helix structure, consisting of “silver-coated glass microspheres/silicone rubber” as the SCT thread and “silicone rubber-coated SCT” as the SSCT thread. Due to the good compatibility of the ultra-stretchable elastomer matrix material, the SATT can easily obtain a high stretchability of 100% to realize conformal assembly in stretchable electronic systems. The SATT with a length of 5 cm generates an output voltage of 3.82 V and output current of 65.8 nA, which could be acted as an active wearable sensor for tracking the finger motion states. Moreover, the SPST woven by the SCT and SSCT units generates the output voltage of 8.1 V and current of 0.42μA in the stretch-release mode and the maximum power can reach up to 163.3 μW in the contact-separation mode. Thus, the SPST is capable to supply the electrical energy for commercial electronics to maintain normal operating state, meanwhile can effectively harvest full-range biomechanical energy from human joint motions, providing a great significance for promoting the development of practical stretchable and wearable energy harvesters.

## Methods

### Fabrication of the SCT

The silver-coated glass microspheres (Shenzhen Xiate Science and Technology Co. Ltd., China) uniformly were dispersed into solid silicone rubber matrix (TN-920) with a weight ratio of 3:1 for 1.5 h. Then, the mixture was placed into a screw extrusion machine to achieve extrusion and vulcanization process at 110 °C and the conductive composite thread with the diameter of 1 mm was obtained. The stretchable five conductive threads were selected to be coiled together and both ends were coated by the mixed silicone rubber (Ecoflex 00-30) and curing agent in a mass ratio of 1:1. Finally, it was placed in a vacuum drying oven evacuated for 20 min and heated for 2 h at 80 °C. After curing and shaping, the SCT could be realized as a stretchable composite electrode.

### Fabrication of the SSCT

The SCT was placed into the mold with 4 mm diameter. Then, the mixture of silicone rubber (Ecoflex 00-30) with curing agent were injected the mold. After evacuating and heating, the SSCT was prepared through the demoulding technology.

### Measurement System

The samples were characterized by field emission scanning electron microscopy (ZEISS EVO18, Carl Zeiss Jena, Germany). The output voltage and current performances were recorded by a KEITHLEY 2611B system electrometer.

## Results and Discussion

**Fig. 1:**
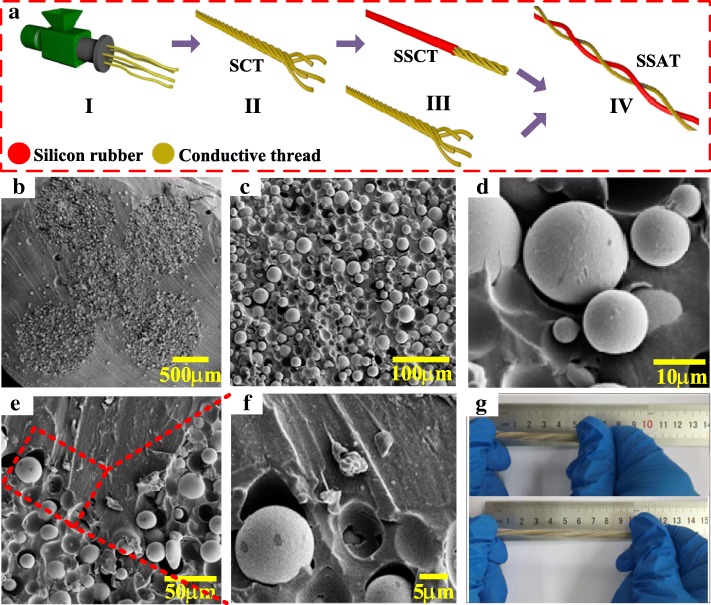
**a** Schematic diagram for fabricating process of the SATT device. **b**–**d** The SEM image of the SSCT cross-section view at different magnifications. **e**, **f** The SEM image of the connection position between SCT and coated silicone rubber at different magnifications. **g** Photographs of the prepared SATT with demonstrations of being stretched at ≈ 100% strain.

The SATT consists of two double-helix all-rubber-based threads: one is the SCT using silver-coated glass microspheres uniformly dispersed into silicone rubber matrix, and the other is the SSCT using the silicone rubber-coated SCT. The detailed fabrication process of the SATT is illustrated in Fig. 1a. The silver-coated glass microspheres (75 wt%) were blended into the ultra-elasticity silicone rubber by mixing process, which was subsequently extruded and vulcanized through the screw extrusion machine to achieve the conductive composite thread (Fig. 1a I). Then the five stretchable conductive threads were selected to be coiled together used as SCT electrode, and the ends of the threads were tied to prevent untwisting during subsequent manufacturing (Fig. 1a (II)). Considering the strong ability to gain electrons, the silicone rubber with superior mechanical property was carefully chosen as a wrapping material to encapsulate the electrode. Namely, the SSCT was prepared and considered as the other triboelectric thread (Fig. 1a (III)). Finally, the SCT and SSCT were intertwined with each other to form a stretchable, wear-resistant, and low-cost all-rubber-based thread-shaped TENG with double-helix structure (Fig. 1a (IV)). The cross-sectional scanning electron microscopy (SEM) image of SSCT is shown in Fig. 1b. It is obvious that the five conductive threads are coated tightly by silicone rubber to achieve an all-in-one structure aiming at more induced charges occurring on the internal conductive threads. As shown in Fig. 1c, d, the silver-coated glass microspheres with different diameters are closely embedded in silicone rubber, which could appear three-dimensional conductive network structure in the rubbery matrix. Consequently, the SCT has an outstandingly conductive property and remarkably stretchable ability. To further demonstrate the good compatibility of homogeneous organic matrix, the SEM images of the enlarged in the connection position between SCT and coated silicone rubber are shown in Fig. 1e, f. Apparently, there is no gap between conductive threads and coated silicone rubber so that they are implemented a well-designed integrated structure. Figure 1g displays the resulted SATT with double-helix energy-scavenging threads, and the lower image of Fig. 1g shows the stretchability of the SATT. The result presents that the thread-shaped TENG can be elongated up to ≈ 100%, which is overwhelmingly superior to the previous reports about thread-based TENG [26–28].

**Fig. 2:**
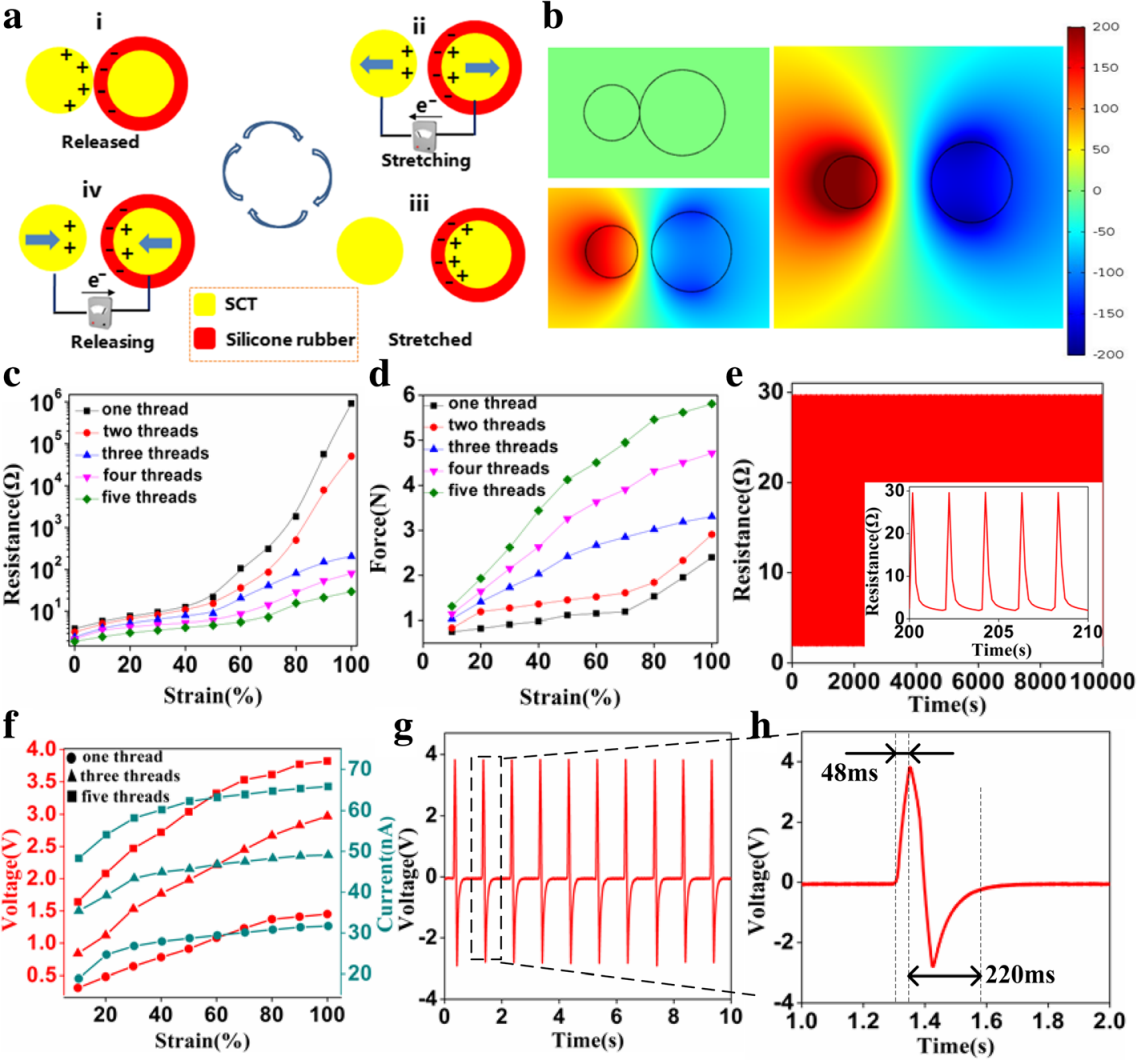
**a** Power generation mechanism of SATT at stretching-releasing process. **b** The simulation results of the potential distributions using COMSOL software. **c** The resistances of conductive threads with the length of 5 cm at different strain mounts. **d** The tensile force experienced by different numbers of conductive threads as functions of strain amount. **e** The tensile durability test of the SCT within 100% strain. **f** The output voltages and currents of conductive threads at different strain mounts. **g** The open-circuit voltage of SATT with a length of 5 cm at 100% strain. **h** Enlarged view of the area indicated by the dashed black box in panel **g**

Despite fabricated by complex double-helix structure, the SATT can be approximated as a large number of capacitors connected in parallel without considering the edge effect. Thus, the working mechanism of SATT could be simplified into the typical contact-separation process between the SCT and SSCT in the stretching-releasing cycles. The electricity-generating mechanism of the SATT based on the coupling effects of contact electrification and electrostatic induction is depicted in Fig. 2a. In the original state, the surface of the silicone rubber takes the negative charges, while an equivalent positive charge is generated on the electrode, respectively, due to the contact electrification. When a tensile stress is applied to the SATT, the distance between the silicone surface and electrode increases and causes an electric potential difference. The electrons flow between two electrodes through the external circuits, resulting in the formation of an electrical current. Until the distance is quite far away, there is an equilibrium state of electrons stopping the transfer. When the tensile stress is released, the electrons flow inversely between the electrodes to realize a charge balance. After the SATT is fully restored to the original state, the charges are completely neutralized again. Thus, the SATT could generate output electrical energy in the continuous stretching-releasing periodic motions.

Furthermore, we establish a finite element method (FEM) simulation based on the COMSOL software to quantitatively analyze the working mechanism of the SATT. In this model, the two tribo-charge densities of ± 1μC/m^2^ are assigned on the thread surfaces. It is worth to note that the amount of initial charges on the thread surfaces only affects the calculated electrical potential; however, the relative changing trend of the electrical potential will be invariant. Figure 2b shows the electrical potential distributions of the SATT at different tensile forces. When the external stretched force does not exist, the potential difference of the whole device is almost zero. As the SATT is stretched outward, the positive and negative tribo-charges are separated, and the potential difference will be increased. Consequently, it is evident that the simulation results by COMSOL software are consistent with the theoretical analysis process of above work mechanism.

For comfortably stretchable electrode, electrical conductivity is an adequately important factor. The proposed stretchable thread-shaped electrode with silver-coated glass microspheres dispersed in silicone rubber elastomer is stretched at different strains to cause varied electric conductivity. It is necessary to systematically study the relationship among the number of conductive threads, the length of the stretch, and the resistance of the electrode. Figure 2c shows the resistances of one to five conductive threads with a length of 5 cm at different strain mounts. Within the range of 50% strain, the resistances of electrodes with different numbers of conductive threads are almost unchanged under the stretching and releasing process. However, with the strain amount increasing, the more numbers of conductive threads, the lower resistance value of the electrode. Figure 2d shows the tensile force experienced by different numbers of conductive threads as functions of strain amount. Obviously, the tensile force will enlarge as the numbers of conductive threads increase. Considering the easier to be stimulated by tensile force, the five intertwined conductive threads are selected as the SCT electrode in this work. The tensile durability of the SCT within 100% strain was performed, as shown in Fig. 2e. The results indicate that the SCT is an excellent conductive elastomer especially exhibiting highly stable reversibility. Additionally, the electrical output performances of the double-helix energy-scavenging threads were carried out, as shown in Fig. 2f. As the increasing number of conductive threads, the contact areas between electrode and silicone rubber are enlarged, resulting in more transferred charges between triboelectric threads under the stretching-releasing motions. Accordingly, both the output open-circuit voltage and short-circuit current increase. Figure 2g presents that the SATT with the length of 5 cm can generate the open-circuit voltage of 3.82 V and the short-circuit current of 65.8 nA at 100% strain. The enlarged view of one voltage cycle is shown in Fig. 2h. It is worthy of note that the response and recovery times of the SATT composed of SCT and SSCT are 48 ms and 220 ms at 1 Hz, respectively. Consequently, the SATT is expected to be used as self-powered tensile sensing electronic to monitor human physiological signals.

**Fig. 3:**
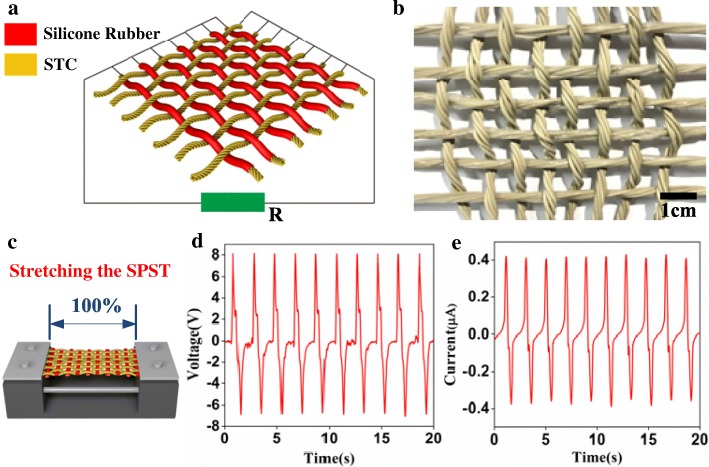
**a** The schematic illustration of the SPST. **b** The photo image of the SPST. **c** The stretching schematic graph of the SPST at the 100% strain. **d** The output voltage and **e** output current of the SPST at the periodic stretching-releasing cycles

The mechanical energy from human motions has been the frequently used energy resources because of its various advantages such as universality, renewability, and stability. Smart textiles and intelligent clothes collecting the mechanical energy from human motions have been widely researched [29–31]. However, due to the lack of excellent stretchability, the comfort of smart textiles based on the flexible strips is an extremely important factor hindering the development of intelligent fabrics. In view of the excellent stretchable characteristic of the SATT device, a lightweight, comfortable, and wearable self-powered textile is put forward here. The SCT and SSCT units were woven into SPST with traditional plain weave. The schematic illustration and photograph of the SPST device (5 × 7 cm^2^) are demonstrated in Fig. 3a, b. It is worth noting that biological movements are normally considered as elongated in 5–30% strain, which requires a much higher strain compatibility of the wearable electronics to ensure long-term stable operation under mechanical tension [32–34]. Figure 3c presents the stretching schematic graph of the intentionally stretched 100% strain of the SPST device using a linear motor. The stretching-releasing working mechanism of SPST is the same as that of SATT that the focus is to connect all STC terminals as the testing port and the electrodes in SSTC together as the other testing port. The open-circuit voltage and short-circuit current of the SPST device are about 8.1 V and 0.42 μA in the process of stretching-releasing excitation, respectively (Fig. 3d, e). Owing to the high stretchability and stable output performances, the SPST could be acted as a self-powered monitoring device to scavenge the stretching kinetic energy for human joints.

**Fig.4:**
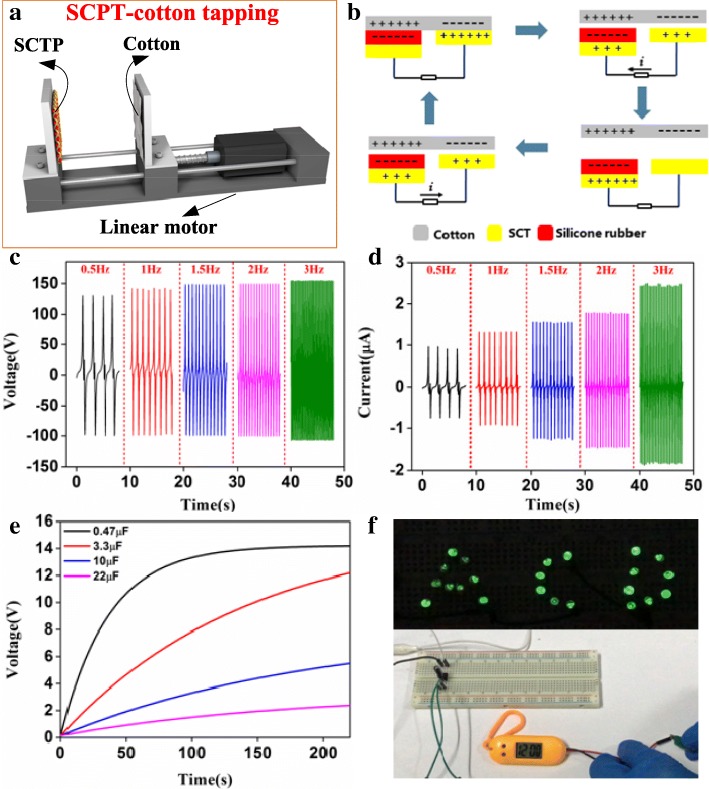
**a** The schematic illustration of the SPST-cotton tapping. **b** The electricity generation mechanism with SPST-cotton tapping. **c** The open-circuit voltages and **d** short-circuit currents with SPST-cotton tapping at different tapping frequencies. **e** Measured voltage curves of various capacitors at frequency of 3 Hz and force of 100 N. **f** The LEDs and electric watch were driven by the SPST-cotton device

Furthermore, considering that the SPST device appears contact-separation process with other clothing fabrics during the actual human movements, the output performances with SPST-cotton tapping were achieved in the periodical tapping process of the linear motor (Fig. 4a). The electricity generation mechanism with SPST-cotton tapping is depicted in Fig. 4b. In the periodical tapping cycles, the contact-separation mode occurs between cotton and the two types of material outside the SPST. Thus, the electrostatic induction charges flow between the electrodes of the SPST. Figure 4c, d displays the open-circuit voltages and short-circuit currents under the force of 100 N. Remarkably, the open-circuit voltage of the SPST is about 150 V at different tapping frequencies, which is independent of the operation frequency. However, the short-circuit currents of the SPST are about 0.96, 1.31, 1.55, 1.77, and 2.45 μA with frequencies of 0.5, 1, 1.5, 2, and 3 Hz, respectively. This is because the time for contact-separation becomes shorter as higher frequencies so that the equal numbers of charges causes a larger current (Isc = dQsc/dt). Furthermore, the SPST acted as an energy supply device usually connects with the external load in practical application. Additional file 1: Figure S1 presents the output voltages as a function of external load resistances from 1 MΩ to 1 GΩ. The output powers of the SPST connected to external loads with various levels can be obtained, since the output power is defined by U^2^/R. Clearly, the output power increases at first and then decreases, reaching a maximum value of 163.3 μW when the external load resistance is about 120 MΩ. In addition, the stability testing of the SPST was conducted for 10,000 cycles, as shown in Additional file 1: Figure S2. Obviously, the SPST’s output voltage did not decline in periodic testing cycles, thus the SPST has the remarkable long lifetime. The electricity generated from the SPST-cotton tapping can be stored into the capacitors to supply power for wearable electronics. Figure 4e shows that the charging curves of various capacities at frequency of 3 Hz and force of 100 N. The voltage of a 0.47 μF capacitor can be charge to 14 V for 150 s. With the capacity of the capacitor increasing, it takes longer to reach the same high voltage. Owing to the outstanding output performances, the SPST-cotton device could directly turn on LEDs and power up a commercial electric watch by the electrical energy stored in the capacitor (Fig. 4f and Additional file 2: Video S1, S2). These results present that the SPST device can provide electrical energy for commercial electronics to maintain normal operation.

**Fig. 5:**
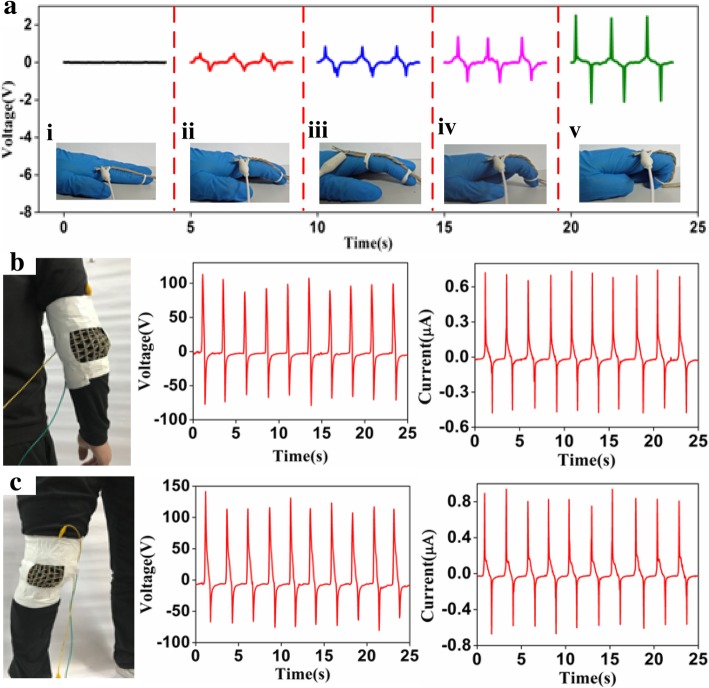
**a** The SATT as a self-powered active sensor for detecting finger motion states. **b** The SPST is fixed on the elbow **c** the knee to perform energy harvesting and condition monitoring

Being stretchable and easy to be assembled in most parts of the body, the thread-shaped TENG can be acted as an active wearable electronic device for detecting the body motions. As shown in Fig. 5a and Additional file 2: Video S3, the SATT device was fixed on a subject’s index figure to respond five bending-releasing motion states. Clearly, the output voltage peaks increase with the enlarging of motion amplitude, namely, the output monitoring signals are determined by the magnitudes of the stretching motions. The behaviors confirm that the SATT can be used as a self-powered active sensor without an external power to quantitatively characterize the finger motion states. Furthermore, the open-circuit voltages of the SPST woven by SCT and SSCT units are stable and independent of the operation frequency, which could be used as the output signals of motion monitoring. As shown in Fig. 5b, c, the SPST was fixed on the joints of human body to perform energy harvesting and condition monitoring. When the flexion and extension behaviors from elbow and knee appear, the stretch-release mode from SPST and the contact-separation mode from SPST-cotton produce, resulting in the alternating electric signals generated. Obviously, the SPST device richly meets the requirement about the elastic property for smart textile, and the output voltages could reach about 105 V and 116.9 V at the maximum bending angles of elbow and knee joints, respectively. The response output currents are about 0.73 μA and 0.89 μA, respectively. Consequently, the carefully designed SPST provides a promising power supply method for wearable electronics by scavenging body joints motion energy and will play an extremely important role in the applications of patients’ rehabilitation training and track activity.

## Conclusion

In summary, this paper demonstrates a newly designed stretchable all-rubber-based thread-shaped wearable electronics by using silver-coated glass microspheres and silicone rubber as source materials. The SATT with 100% strain can convert tensile mechanical energy into electric energy via electrostatic effect and is demonstrated as a self-powered sensor to quantitatively track the finger joint motions. Moreover, the SCT and SSCT triboelectric threads are woven into SPST with traditional plain weave, which generates the open-circuit voltage of 8.1 V and short-circuit current of 0.42 μA through the stretching-releasing interaction between knitting units and the maximum output power of 163.3 μW at external load resistance of 120 MΩ in the SPST-cotton tapping way. With the stable and large output voltage performance, the SPST had been identified as an effective power source to supply the electrical energy for commercial electronics. Being stretchable and wearable, the SPST provides an effective solution for harvesting biomechanical energy from human joint motions and will be expected to develop great potential in the applications of in medical systems and self-powered smart tracking technologies.

## Additional files

Additional file 1: **Figure S1.** The output voltages as a function of external load resistances from 1 MΩ to 1 GΩ. **Figure S2.** The output performances stability testing of the SPST is conducted for 10,000 cycles. (DOCX 15855 kb)

Additional file 2: **Video S1.** The SPST-cotton device can directly turn on LEDs. **Video S2.** The SPST-cotton device can power up a commercial electric watch by the electrical energy stored in the capacitor. **Video S3.** The SATT device was fixed on a subject’s index figure to respond five bending-releasing motion states. (ZIP 11240 kb)

## Data Availability

All data generated or analyzed during this study are included in this published article and its supplementary information files.

## Abbreviations

SATT: Stretchable all-rubber-based thread-shaped TENG
SCT: Stretchable conductive thread
SEM: Scanning electron microscopy.
SPST: Self-powered smart textile
SSCT: The silicone rubber coated SCT
TENG: Triboelectric nanogenerator
