# Supplementary material for: Highly Stretchable All-Rubber-Based Thread-Shaped Wearable Electronics for Human Motion Energy-Harvesting and Self-Powered Biomechanical Tracking
Source: Nanoscale Res Lett. 2019 Jul 23;14:247. doi: 10.1186/s11671-019-3085-9 (PMC6650513; doi:10.1186/s11671-019-3085-9)
Supplement: Supplementary file 1 — Figure S1. The output voltages as a function of external load resistances from 1 MΩ to 1 GΩ. Figure S2. The output performances stability testing of the SPST is conducted for 10,000 cycles. (DOCX 15855 kb) [file 11671_2019_3085_MOESM1_ESM.docx]

**Supporting Information**

**Highly stretchable all-rubber-based thread-shaped** **wearable electronics for human motion energy harvesting and** **self-powered biomechanical tracking**

Jie Zhu^1*^, Xinghui Wang^1^,Yilan Xing^1^, Jianyi Li^1^

1 School of Computer and Remote Sensing Information Technology, North China Institute of Aerospace Engineering, Langfang 065000,China;

Correspondence: [zhujie0424@126.com](mailto:zhujie0424@126.com);





**Figure S1.** The output voltages as a function of external load resistances from 1MΩ to 1GΩ.





**Figure S2.** The output performances stability testing of the SPST was conducted for 10000 cycles.
